# Relationship Between Mobile Digital Sensor Monitoring and Perioperative Outcomes: Systematic Review

**DOI:** 10.2196/21571

**Published:** 2021-02-25

**Authors:** Ali Memon, Patrick Lec, Andrew Lenis, Vidit Sharma, Erika Wood, George Schade, Wayne Brisbane

**Affiliations:** 1 Department of Urology University of Washington Seattle, WA United States; 2 Department of Urology University of California, Los Angeles Los Angeles, CA United States

**Keywords:** mobile sensors, perioperative, sensor monitoring, perioperative outcomes

## Abstract

**Background:**

Monitoring surgical recovery has traditionally been confined to metrics measurable within the hospital and clinic setting. However, commercially available mobile sensors are now capable of extending measurements into a patient’s home. As these sensors were developed for nonmedical applications, their clinical role has yet to be established. The aim of this systematic review is to evaluate the relationship between data generated by mobile sensors and postoperative outcomes.

**Objective:**

The objective of this study is to describe the current use of mobile sensors in the perioperative setting and the correlation between their data and clinical outcomes.

**Methods:**

A systematic search of EMBASE, MEDLINE, and Cochrane Library from inception until April 2019 was performed to identify studies of surgical patients monitored with mobile sensors. Sensors were considered if they collected patient metrics such as step count, temperature, or heart rate. Studies were included if patients underwent major surgery (≥1 inpatient postoperative day), patients were monitored using mobile sensors in the perioperative period, and the study reported postoperative outcomes (ie, complications and hospital readmission). For studies including step count, a pooled analysis of the step count per postoperative day was calculated for the complication and noncomplication cohorts using mean and a random-effects linear model. The Grading of Recommendations, Assessment, Development, and Evaluation tool was used to assess study quality.

**Results:**

From 2209 abstracts, we identified 11 studies for review. Reviewed studies consisted of either prospective observational cohorts (n=10) or randomized controlled trials (n=1). Activity monitors were the most widely used sensors (n=10), with an additional study measuring temperature, respiratory rate, and heart rate (n=1). Low step count was associated with worse postoperative outcomes. A median step count of around 1000 steps per postoperative day was associated with adverse surgical outcomes. Within the studies, there was heterogeneity between the type of surgery and type of reported postoperative outcome.

**Conclusions:**

Despite significant heterogeneity in the type of surgery and sensors, low step count was associated with worse postoperative outcomes across surgical specialties. Further studies and standardization are needed to assess the role of mobile sensors in postoperative care, but a threshold of approximately 1000 steps per postoperative day warrants further investigation.

## Introduction

Of the 234.2 million patients who undergo major surgery every year globally, around 7 million will experience a postoperative complication [1]. These events are difficult for patients and clinicians and costly for hospitals and third-party payers [2]. Enhanced recovery after surgery (ERAS) protocols have reduced complication and readmission rates among various surgical specialties [3,4]. ERAS protocols assist patients to return to their preoperative baseline by minimizing opiate analgesia, promoting nutrition, and encouraging early ambulation. This has been shown to speed recovery and avoid hospital-acquired infections and complications [5]. However, these programs rely on interventions within hospitals and clinics and thus fail to extend recovery measurements into a patient’s home.

It is now possible to track patient health metrics using commercially available mobile sensors. It may be possible to use these sensors to optimize patient recovery outside of the hospital. Commercial sensors are ubiquitous and relatively inexpensive, with almost 20% of American adults owning one [6]. Among the many options for sensor output, it is unclear which metrics are most clinically useful or hold promise for future research. For example, step count monitors are common and physical activity has been tied to favorable postoperative outcomes [7,8]. However, it is difficult to know exactly how step count modifies variables other than length of stay [9,10] or if length of stay can serve as a surrogate for impending complications [11]. As investigators seek to incorporate mobile sensors into ERAS pathways, there is a need for a synthesized evaluation of how mobile sensor output data correlate to postoperative outcomes.

Synthesizing the current literature may shed light on the validity of employing these devices in future prospective studies and guide efforts to improve perioperative care. While another recent meta-analysis reported the association between perioperative mobile sensor step count and length of stay, no study has compiled data on other important perioperative outcomes, such as 30- and 90-day morbidity and mortality and readmission [7]. Therefore, the aim of this systematic review is to describe the current use of mobile sensors in the perioperative setting and the correlation between their data and clinical outcomes.

## Methods

### Literature Search

A literature search was performed using MEDLINE, EMBASE, and the Cochrane Library from inception until April 2019 to identify studies of surgical patients monitored with mobile sensors. Searches included whole-field terms without quotations to maximize results. Medical subject headings for the literature search included surgery (subheading), activity tracker, and heart rate. A total of 13 search combinations were used, with “surgery” as the anchor followed by one or more of the following search terms: activity, tracker, fitness, heart, rate, Fitbit, Garmin, Apple Watch, Actigraph, Misfit, Huawei, Moov, Motiv, Sensewear, Omron, and wearable. Two reviewers (AM and WB) independently reviewed abstracts for inclusion and exclusion criteria and identified studies for full-text manuscript review. Manuscript review was performed independently, and disagreements in article selection for qualitative analysis were resolved by discussion or by a third blinded reviewer (GS). Although the literature search was conducted over a year prior to the publication of this systematic review, we believe that it is likely not an issue for the validity of the data, considering that many Cochrane reviews have been found to take similar lengths of time for submission, highlighting the lengthy process associated with submitting a systematic review [12]. 

### Inclusion Criteria

Only studies reporting on major surgery, defined as any surgery requiring a postoperative hospitalization of at least one day, were included. Patients were required to have perioperative measurements with a mobile sensor and correlative clinical outcome measures reported. Any sensor type was included, provided it was mobile and capable of evaluating patients while at home. We did not require any particular reporting mechanism for documenting complications, such as the Clavien-Dindo classification. Both randomized trials and observational cohorts were included. While important, studies with a primary outcome of hospital length of stay were not included, as this outcome has been addressed in a recent meta-analysis [7]. The search was restricted to English-language articles.

### Exclusion Criteria

Upon full-text review, studies were excluded if they evaluated patients younger than 18 years, sensors were not mobile (eg, continual electrocardiogram with a stationary receiver), patients were discharged without hospitalization, they lacked reported perioperative outcomes (ie, only reporting sensor output over time without a correlated perioperative outcome), or they represented a redundant patient population from an earlier study. Meta-analyses, case studies, and conference abstracts without an associated manuscript were reviewed for primary references but not directly included.

### Study Quality

The quality of each selected article was assessed using the Grading of Recommendations Assessment, Development, and Evaluation (GRADE) system [13]. The GRADE system classifies the quality of evidence into high, moderate, low, or very low. This tool can be applied to both randomized controlled trials and observational studies. Disagreements between the reviewers (AM and WB) over the risk of bias were resolved by discussion or with involvement of a third reviewer (GS).

### Data Extraction

Data from the reviewed manuscripts were extracted according to the PICO (population, intervention, comparison, and outcome) model for data extraction [14] using a standardized template modified to capture information regarding perioperative complications and sensor information [2]. The following data were extracted: (1) study features, including study design, type of surgery, number of patients, and patient demographics; (2) mobile sensor details, including type of sensor and sensor output measures (eg, number of steps, energy expenditure); (3) author-defined surgical outcomes, including readmission, 30- and 90-day complications, skilled nursing facility (SNF) discharge, and mortality. 

## Results

### Search Results and Study Selection

Our literature search identified 2199 records using the protocol described above. An additional 10 manuscripts were identified by review of references. Using a priori exclusion criteria, 2130 records were removed, as shown in Multimedia Appendix 1. We evaluated 79 full-text articles, of which 11 manuscripts met inclusion criteria. The most common reason for manuscript exclusion was failure to report perioperative surgical outcomes (n=44). All authors whose studies used accelerometers were contacted by email up to two times to obtain patient-level step count and complications for meta-analysis. One author replied to our request but could not provide these data secondary to the interval since the study’s completion. Due to the lack of patient-level data, meta-analysis of step count and complication rate was not performed. A forest plot was completed with the limited step count data available from 4 studies, and its results are summarized in Multimedia Appendix 2. Prior to performing a literature search, the study protocol was prospectively registered with the PROSPERO (International Prospective Register of Systematic Reviews) international database of systematic reviews (ID No. CRD42020134656).

### Study Aims and Outcomes

As reported in Multimedia Appendix 3, a total of 10 of 11 studies evaluated physical activity. Of these, 7 specified the evaluation of activity and perioperative outcomes as a specific aim [15-21]. A total of 3 studies evaluated complications but did not mention if it was a primary or secondary end point [22-24]. One study evaluated temperature, heart rate, and respiratory rate as they related to interventions for sepsis and overall survival along with the secondary outcome of readmission [25].

### Study Characteristics

This review encompassed 949 patients with activity data (step count: n=838; other: n=111). The included studies spanned publication from 2007 to 2019. Of the 11 surgical cohorts, 3 consisted of cardiothoracic cases (coronary artery bypass graft or coronary valve replacement: n=2; pulmonary wedge resection: n=1). A total of 6 studies evaluated patients recovering from general surgery procedures, including gastrointestinal surgery, colorectal surgery, hepatic resection, esophagectomy, and peritoneal cancer debulking with heated intraperitoneal chemotherapy. Finally, 1 study followed patients after lumbar spine fusion, and 1 evaluated patients following open vascular bypass grafting of the extremities. The largest study cohort consisted of 226 patients [25], while the smallest included 11 patients [16]. Surgical outcome variables were author defined and included Clavien-Dindo graded surgical complications, pulmonary and cardiovascular complications, sepsis, hospital readmission, and SNF discharge. A summary of study characteristics is available in Multimedia Appendix 3.

### Sensor Characteristics

Sensors were located on the wrist (n=3), hip (n=3), thigh (n=2), upper arm (n=1), ankle (n=1), and chest (n=1). Within the 10 studies measuring physical activity, outputs included steps (n=6), length of time being physically active or upright (n=3), and daily caloric expenditure (n=1). A single study used a sternal sensor to record temperature, heart rate, and respiratory rate [25].

### Physical Activity Output

As reported in Multimedia Appendix 4, a total of 9 of the 11 studies recorded physical activity using data from a triaxial accelerometer. Output from the accelerometer was reported as step counts in 6 of 9 studies. Alternatively, 3 of 9 studies reported accelerometer output as metabolic equivalents or time spent in activity. The earliest published study utilized an in-house uniaxial accelerometer that provided the duration of time that the patient was lying supine versus upright. When combining the reported mean step count per postoperative day for the 6 studies providing these data, we found that the average step count associated with postoperative complications was a mean of 1099 (SD 561) steps, while the step count associated with recovery without perioperative outcomes was a mean of 2184 (SD 1090) steps.

We performed a meta-analysis of how step count related to complications using a random-effects linear model adjusting for study size. A total of 4 of the 6 studies reporting mean step counts were included. Adjusting for number of participants, patients in the high-complication cohorts took 963 fewer daily steps than those in the low-complication cohorts (*P*=.38), as shown in Multimedia Appendix 1.

### Non–Physical Activity Output

A single study evaluated temperature, heart rate, and respiratory rate for continuous vital sign measurements after surgery [25]. This system of evaluation was found to be superior to standardized vital sign measurements to inform an early warning score [26]. This resulted in earlier identification of sepsis and improved time to antibiotic administration, shortened length of stay, and decreased 30-day readmission rate.

### Study Quality

Of the included studies, 8 were rated as low quality by GRADE criteria secondary to major differences between surgical groups, low participant number, high variability in results, and perception of bias (Multimedia Appendix 3) [13,15-20,22,23]. Two studies were rated as moderate quality due to medically homogenous participants and the lack of preoperative ambulatory information [21,24]. One study was rated as high quality due the results being obtained in a well-designed randomized controlled trial [25].

## Discussion

### Summary of Findings

This systematic review evaluated surgical patients monitored with a mobile sensor and correlated sensor output with postoperative outcomes. We identified 11 studies, of which 10 used physical activity tracking, with steps being the most common sensor output. There was heterogeneity between studies, yet our systematic review of the published data shows that lower step count was associated with increased adverse postoperative outcomes. This will require further prospective validation, but our evaluation suggests that a step count cutoff of around 1000 steps per day may be prognostic. We found limited evidence for other mobile sensors, such as heart rate, respiratory rate, and temperature sensors. However, the available data are promising. Going forward, an effort is needed to standardize reporting for mobile sensor output and postoperative outcomes to aid integration into ERAS protocols and the development of prognostic markers.

It has long been postulated that higher levels of postoperative activity are associated with improved outcomes. It is also likely that baseline activity represents a surrogate marker of overall patient health. Prior evaluation of physical activity and length of stay demonstrated that increased physical activity was associated with decreased length of stay [9]. It is likely that mobile digital sensors can also improve postoperative recovery as an adjunct to ERAS protocols by providing continuous patient data that providers can use to intervene early before complications occur [27]. Such sensors enable providers to monitor patients after the patient has departed from the hospital. There is early evidence that implementing home monitoring can result in postoperative improvement. However, it remains unclear which sensors and outputs should be the focus of such programs [28]. It is likely that a surgery-specific step count would be able to produce better predictions on patients needing enhanced recovery care [10]. Our study demonstrates that increased activity is associated with a broad set of improved postsurgical outcomes. In its current state, this information can inform postoperative risk stratification and with future work could be integrated into ERAS protocols.

While the benefits of activity are consistent with clinical experience, it is unclear to what extent these findings can be clinically implemented. We found that step counts of around 1000 steps per day are associated with adverse surgical outcomes. We hypothesize that in future studies, a step count below 1000 steps per day will be specific for a high risk of complication, while step counts greater than 2000 steps per day will be sensitive for ruling out complications. Such thresholds could inform when patients should be assigned more intensive postoperative care or be allowed early discharge.

Although physical activity represents an objective metric for health, an important limitation of this study is the broad inclusion of outcome definitions. Prior work has focused on length of stay, as it offers a common metric for postoperative recovery [9]. However, length of stay does not necessarily correlate with postoperative outcomes [11]. In fact, there is concern that shortening length of stay for certain surgeries may increase adverse events once the patient returns home [29]. A barrier to implementation will be the heterogeneity in sensor types [30,31]. This was well demonstrated in our evaluation, as the only 2 studies to use the same sensor involved the same author and institution [19,21]. However, despite differences in sensor type and outcomes, there were generally more favorable postsurgical outcomes with increased activity and continuous monitoring. Future studies should standardize reporting and possibly the type of sensors used in order to strengthen the pooled data analysis. It is likely that surgical specialties will need to define the most salient outcomes, but they must also report on standard outcomes, such as readmission and 30- and 90-day mortality rates.

Taken as a whole, this summary identifies that low activity and step count are associated with a heterogenous increase in adverse outcomes. Additionally, there is high-quality evidence that continuously tracking heart rate, respiratory rate, and temperature is also useful for early identification of sepsis. Mobile sensors will increasingly be implemented into postoperative convalescence in an effort to improve surgical outcomes. Such implementation has the potential to provide granular patient data on health and recovery before and after the index hospitalization. A major clinical barrier that merits considerable research is which sensors should be used and which output thresholds warrant clinical concerns. With sensor standardization and integration into the electronic health records, there is a potential to create predictive algorithms with considerable predictive power [32]. However, there will also be barriers to integrating these predictions into the clinical workflow [33]. Going forward, clinicians and industries should partner to validate how commercially available sensors can augment patient and clinician decision making. Additionally, collaborative efforts, such as those suggested by the mobile sensor data-to-knowledge program, should be used by investigators to pool resources and improve data and usability [34].

The main limitation of this study is that the number of studies that make up the systematic review is small and that within this review, 8 of 11 studies were low quality when using the GRADE tool, which is likely a reflection of the nascent state of this investigation but also underscores the potential for future study. While we were not able to find a statistically significant difference between step count and postoperative complications, we did find an association between lower step count and lower postoperative complications, which needs prospective evaluation. Additionally, the mean step counts associated with postoperative complications or recovery were quite different, and the fact that step count is a continuous variable should not completely discredit the merits of our findings.

### Conclusions

Digital mobile sensors enable real-time postoperative monitoring of step count, activity, heart rate, respiratory rate, and temperature and have now been used in initial clinical studies. While significant heterogeneity between sensor type, measured output, and reported outcomes limit the generalizability and interpretation of the presented body of literature, several studies successfully demonstrate the potential for mobile sensors to measure clinically relevant metrics. High-quality prospective studies are required to establish the most clinically relevant metrics and threshold values to incorporate into care algorithms.

## Appendix

Multimedia Appendix 1 Study selection process.

Multimedia Appendix 2 Forest plot utilizing available step count data from four studies.

Multimedia Appendix 3 Characteristics of the studies included.

Multimedia Appendix 4 Mobile sensor monitoring characteristics for the studies included.

## Abbreviations

ERAS: enhanced recovery after surgery
GRADE: Grading of Recommendations Assessment, Development, and Evaluation
PICO: population, intervention, comparison, and outcome
PROSPERO: International Prospective Register of Systematic Reviews
SNF: skilled nursing facility
