# Supplementary figures and images for: Relationship Between Mobile Digital Sensor Monitoring and Perioperative Outcomes: Systematic Review
Source: JMIR Perioper Med. 2021 Feb 25;4(1):e21571. doi: 10.2196/21571 (PMC7952235; doi:10.2196/21571)

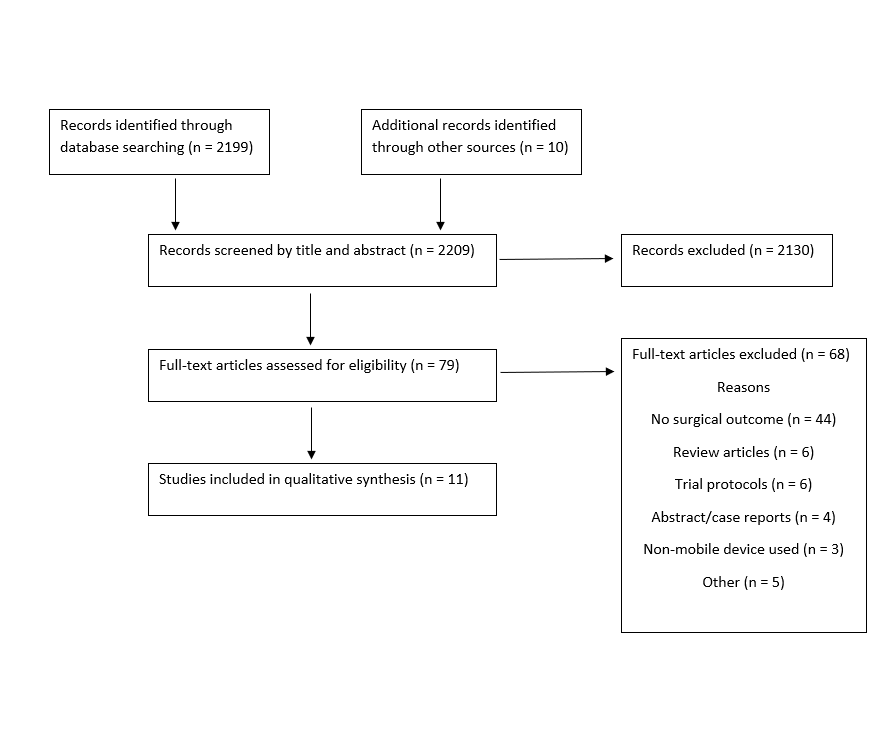

Supplement: Multimedia Appendix 1 [file periop_v4i1e21571_app1.png]

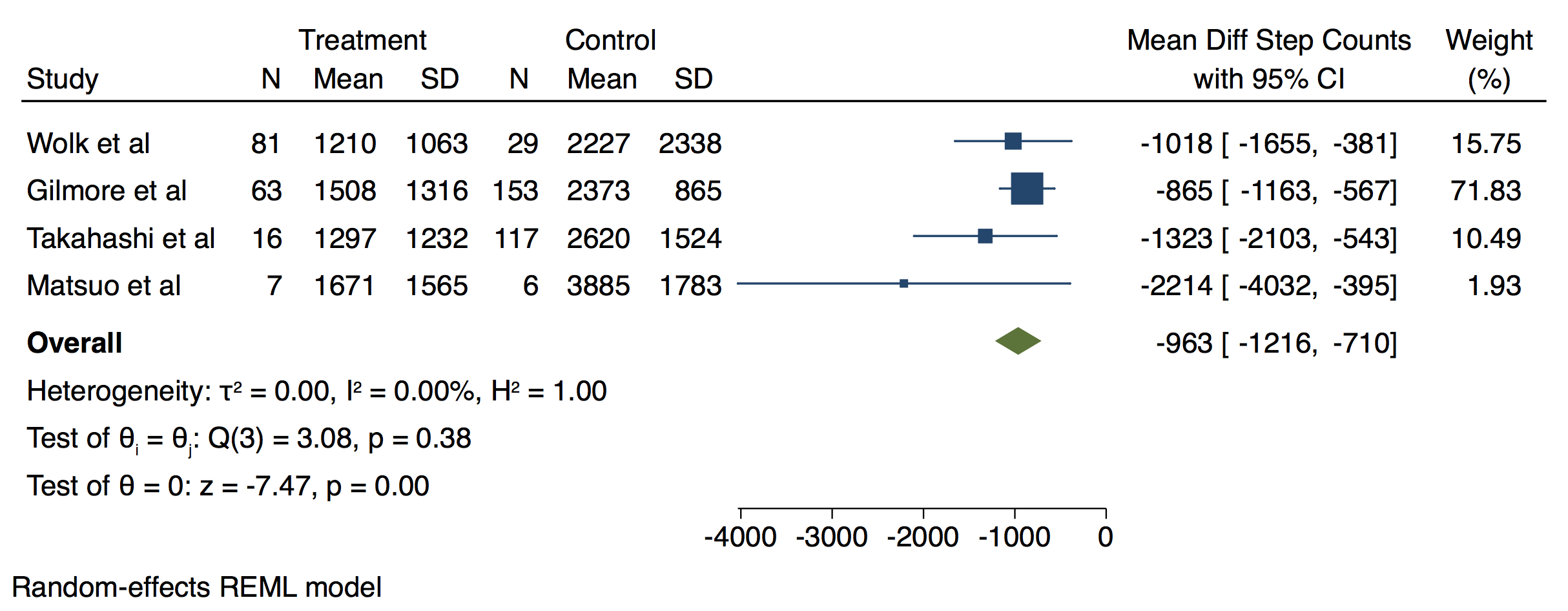

Supplement: Multimedia Appendix 2 [file periop_v4i1e21571_app2.png]

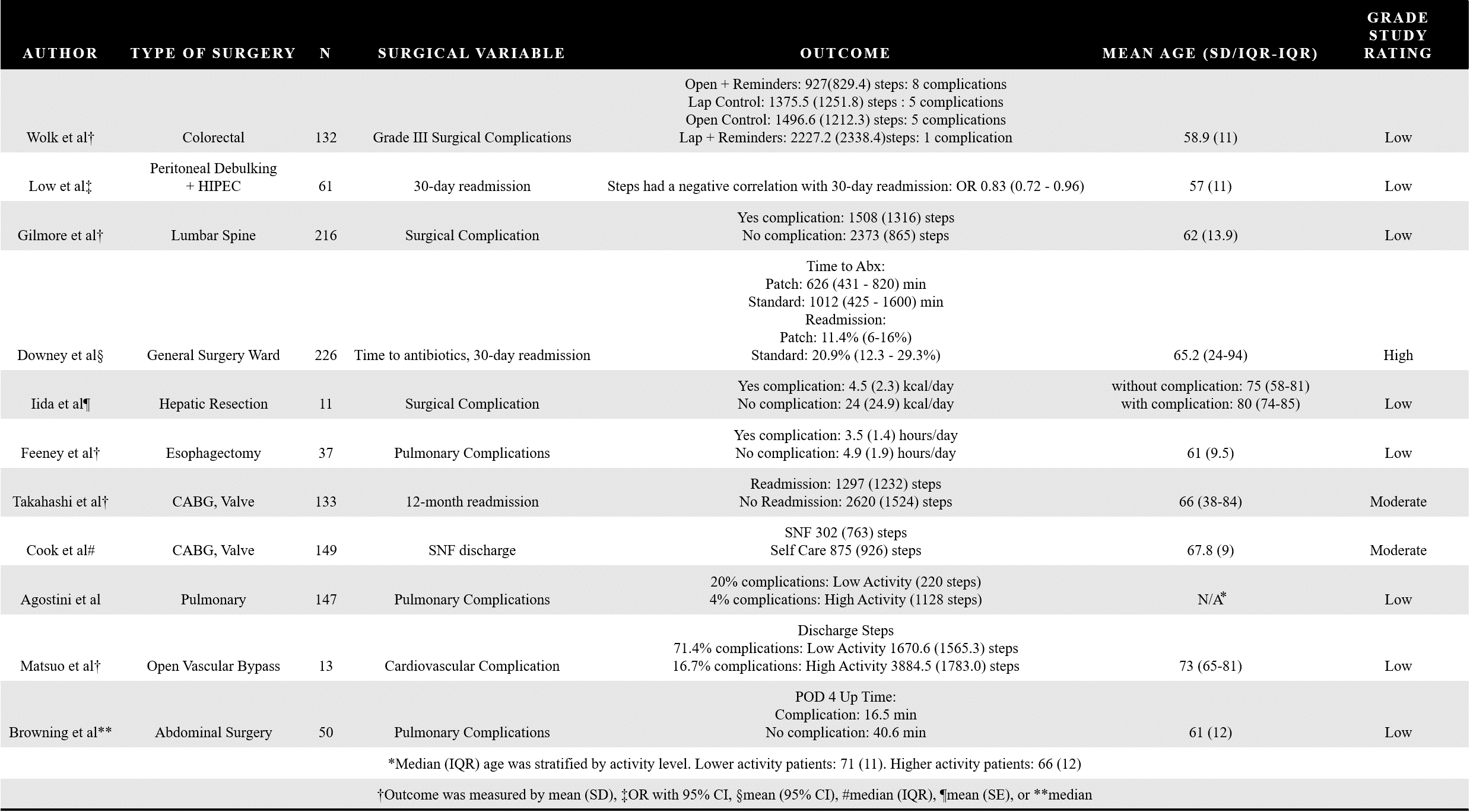

Supplement: Multimedia Appendix 3 [file periop_v4i1e21571_app3.png]

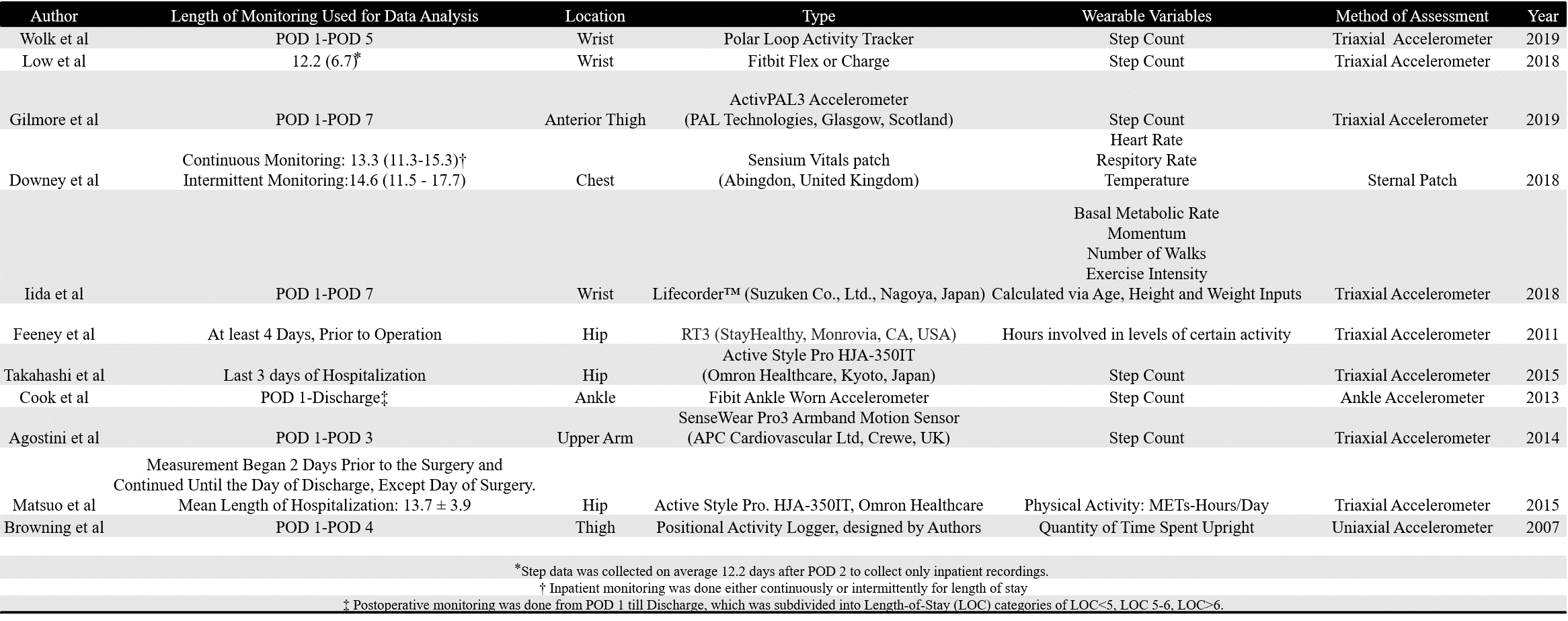

Supplement: Multimedia Appendix 4 [file periop_v4i1e21571_app4.png]
